# Coordination of anti-CTLA-4 with whole-brain radiation therapy decreases tumor burden during treatment in a novel syngeneic model of lung cancer brain metastasis

**DOI:** 10.1007/s00262-023-03599-w

**Published:** 2024-01-19

**Authors:** K. E. Blethen, C. P. Wolford, G. L. Pecar, T. A. Arsiwala, E. Adjeroh, L. P. Dykstra, B. N. Kielkowski, P. R. Lockman

**Affiliations:** 1 Department of Pharmaceutical Sciences, School of Pharmacy, West Virginia University, 108 Biomedical Drive, Morgantown, WV 26506 USA; 2 Rockefeller Neuroscience Institute, West Virginia University, 1 Medical Center Dr, Morgantown, WV USA

**Keywords:** Immunotherapy, Immune checkpoint blockade, Brain metastasis, EGFR, Radiotherapy

## Abstract

Lung cancer is the most common primary tumor to metastasize to the brain. Although advances in lung cancer therapy have increased rates of survival over the past few decades, control and treatment of lung cancer brain metastasis remains an urgent clinical need. Herein, we examine the temporal coordination of α-CTLA-4 administration in combination with whole-brain radiation therapy in a syngeneic preclinical model of lung cancer brain metastasis in both C57Bl/6 and athymic nude mice. Brain tumor burden, survival, and weight loss were monitored. Immunotherapy administration 24 h prior to irradiation resulted in increased brain tumor burden, while administration of immunotherapy 12 h after radiation decreased tumor burden. Neither of the treatments affected survival outcomes or weight loss due to brain tumor recurrence. These findings suggest that the coordination of α-CTLA-4 administration in addition to whole-brain radiation therapy may be a viable strategy for reduction of tumor burden for the management of lung cancer brain metastasis.

**Supplementary Information:**

The online version contains supplementary material available at 10.1007/s00262-023-03599-w.

## Introduction

Lung cancer is the leading cause of cancer deaths globally and is among the most common cancer types to result in brain metastasis, with approximately 40% of patients developing brain metastases in their lifetime. While innovations in lung cancer therapy in recent decades have increased 5-year survival from 14 to 24%, the 5-year survival rate for patients with lung cancer brain metastases (LCBM) remains at 4.7% [1, 2]. These data highlight an urgent need for new approaches to the management of LCBM. Currently, approved therapies for brain metastases include stereotactic radiosurgery (SRS), whole-brain radiation therapy (WBRT), surgical resection, chemotherapies, and targeted therapies. Typically, patients will receive varied combinations of treatments to manage the primary tumor and resulting metastases.

Although immunotherapy has been successful in the management of primary lung tumors [3–5], evidence for the efficacy of immunotherapy for LCBM is mostly limited to retrospective analyses due to the frequent exclusion of patients with brain metastases from clinical trials [6–8]. Nevertheless, the few available prospective clinical trials seem promising [9, 10]. For example, a small phase II study in patients with previously untreated brain metastases resulted in an intracranial response rate of 29.7% following treatment with pembrolizumab [NCT02085070]. Additional prospective clinical trials are needed to validate the intracranial efficacy of immunotherapies.

The optimal sequence of immunotherapy administration and radiotherapy has not yet been characterized. Peripheral tumor biology demonstrates the rationale for combining radiotherapy and immunotherapy to produce synergistic effects, presumably due to the abscopal response elicited by radiation [11, 12]. A recent phase I/II clinical trial evaluated the safety and efficacy of nivolumab and ipilimumab with concurrent SRS in patients with LCBM. The study concluded that the combinatorial therapy had minimal toxicity and a 4-month progression-free survival rate of 70.7% [13]. However, it is still unclear which treatment should be administered first, and whether the therapeutic sequence is a determinant of patient response. An additional challenge to LCBM immunotherapy research is a lack of appropriate preclinical models, with most established models utilizing human cancer cells in immunocompromised animals [14].

We have previously demonstrated an inflammatory response and breakdown of the blood–brain barrier 12 h following whole-brain radiation therapy (WBRT) in wild-type C57Bl/6 mice [15]. Due to the aforementioned lack of syngeneic LCBM models, we developed and characterized an immunocompetent preclinical model of LCBM to evaluate immunotherapy efficacy. We hypothesized that administration of immunotherapy post-WBRT would decrease tumor burden and increase survival. The α-CTLA-4 immunotherapy was selected due to its well-documented mobilization of T-cells to “cold” tumors, elicitation of clonal diversity, rare tumor recurrence following treatment, and efficacy in various preclinical and clinical studies when combined with radiotherapy [16–18].

This study aimed to 1) develop a syngeneic model of LCBM and 2) evaluate if timed administration of immunotherapy with radiotherapy increases survival and decreases tumor burden. Herein, we successfully generated a syngeneic LCBM preclinical model amenable for use in immunocompetent and immunocompromised mice. Mice were treated with clinical radiotherapy dosing schedule of 30 Gy in 10 fractions delivered over 12 days. We hypothesized that immunotherapy would be more efficacious when delivered 12-h post-WBRT than if it was delivered 24 h prior to WBRT. Administration of immunotherapy 12 h after WBRT significantly decreased tumor burden in immunocompetent mice compared to administration of immunotherapy 24 h prior to radiation. Unexpectedly, the responses to immunotherapy and radiation were not durable, and treatment had no effect on survival. Together these data suggest that the appropriate timing of immunotherapy administration relative to radiotherapy is important to delay tumor progression, but treatments may need to be continued long term, and use of immunotherapy for brain metastasis warrants further study.

## Materials and methods

### Cell culture

The parental LLC-Luc2 cell line was purchased from ATCC (Manassas, VA) and came transduced to express firefly luciferase to allow for bioluminescence imaging (BLI). Cells were cultured in DMEM (ATCC, Manassas, VA) supplemented with 10% fetal bovine serum (Global Life Sciences Solutions, Cranbury, NJ), 1% antibiotic–antimycotic (Thermo Fisher Scientific, Waltham, MA), and 2 µg/mL blasticidin (Thermo Fisher Scientific, Waltham, MA) to ensure the selection of transduced cells. Cells were incubated at 37 °C and 5% CO_2_. All cells used for in vivo and in vitro experiments were maintained between passages 1–8.

### Scratch assay

A 24-well plate was coated with collagen (Sigma-Aldrich, St. Louis, MO) at a concentration of 100 µg/mL and placed in a refrigerator at 4 °C. The parental (LLC-P) and brain-tropic (LLC-Br) cell lines were plated at 5 × 10^5^ cells/well and incubated overnight at 37 °C and 5% CO_2_. The following day, a scratch was made in each well with a 200-µL pipette tip and imaged on an Olympus MVX stereomicroscope (Olympus, Tokyo, Japan) (optical zoom range 0.63–12.6, NA = 0.5) immediately, 1-, 2-, 3-, 6-, 12-, and 24-h post-scratch. A wound healing application on ImageJ software was utilized to calculate scratch area of each image. The area of the scratch at each time point was divided by the area at T_0_ and multiplied by 100 to calculate the percent wound closure over time.

### Animals and brain tumor model development

All animal experiments were approved by the Institutional Animal Care and Use Committee at West Virginia University. Female C57Bl/6 and athymic nude mice were purchased from Jackson Laboratory (Bar Harbor, ME). All animals were approximately 6–8 weeks of age and ~ 23 g during tumor implantation. Animals were allowed to acclimate for at least 1 week prior to experimentation. Mice were anesthetized with 2% isoflurane and placed into a stereotactic device (Stoelting, Wood Dale, IL). Animals were injected with 150,000 LLC cells suspended in 100-µL PBS in the left ventricle of the heart. Biweekly BLI was performed to confirm tumor presence in the brain. The protocol for the development of the brain-seeking line was modified from Yoneda et al. and previously described by our laboratory [19, 20]. Animals were euthanized once we observed significant BLI signal, and brains were collected then homogenized and digested with collagenase in DMEM. The homogenate was ejected from a 19G needle and strained with a 70-µm cell strainer to produce a single-cell suspension. The solution was centrifuged and resuspended three times with DMEM and 50% FBS, PBS, and then 25% BSA in PBS to remove the myelin layer. The remaining cell pellet was resuspended in complete medium and cultured. Once the flask became confluent, cells were washed with PBS thrice and injected into mice. This process was repeated until cells predominantly seeded into the brain, which was six times for the LLC cell line, referred to as LLC-Br.

### Monitoring tumor progression, weight, and survival

Tumor progression and weight were monitored biweekly. Animals were injected intraperitoneally with 150 mg/kg D-luciferin potassium salt (PerkinElmer, Waltham, MA) and anesthetized with 2–3% isoflurane. Approximately 15-min post-injection, animals were imaged with the IVIS Spectrum CT (PerkinElmer, Waltham, MA), and bioluminescence (BLI) was captured at auto-exposure and 1-min time frames on Stage D with medium binning. BLI was quantified by drawing a region of interest (ROI) around the cranium or peripheral body of each mouse. The BLI is reported as radiance (photons/sec/cm^2^/steradian). The brain-to-body ratios were calculated by dividing radiance of the brain ROI by the radiance of the peripheral body ROI. Fold change of brain BLI was normalized to radiance at day 3. Mice were euthanized when they displayed signs of neurological symptoms or had over 20% weight loss. Survival was monitored and plotted on a Kaplan–Meier curve.

### Irradiation protocol

As described previously, the XenX irradiator (Xstrahl, Suwanee, GA) at West Virginia University was commissioned to deliver clinically-relevant doses of radiation [21]. On day 3, mice were randomized into five groups (vehicle, α-CTLA-4 only, radiation only, α-CTLA-4 24 h before WBRT, and α-CTLA-4 12-h post-WBRT) and began treatment. Mice receiving radiation therapy were anesthetized with 1–3% isoflurane and treated with whole-brain irradiation at a dose of 30 Gy in 10 fractions delivered over 12 days, the clinical radiation treatment schedule.

### Immunotherapy preparation and administration

The anti-CTLA-4 antibody (Bio X Cell, Lebanon, NH) was diluted in Bio X Cell’s recommended InVivoPure dilution buffer (pH 7.0) to deliver 100 µg of antibody/100 µL intraperitoneally. Immunotherapy treatment groups began treatment on day 3 and were treated twice more on days 6 and 9. The vehicle group received mouse IgG2b isotype control antibody (Bio X Cell, Lebanon, NH) diluted 100 µg/100 µL in dilution buffer and delivered on the same days immunotherapy groups received treatment. Immunotherapy groups combined with radiation were delivered their treatment either 24 h prior to radiation or 12 h after.

### Statistical analysis

Data were analyzed and plotted with GraphPad Prism 8 software (GraphPad Software, San Diego, CA). Results are presented as mean ± S.E.M. unless noted otherwise. Statistical differences between two groups were assessed using Student’s t-test. One-way ANOVA with a Tukey post-test was utilized for data with more than two groups. Differences were considered statistically significant at *p* < 0.05 (*).

## Results

### Development of the brain-tropic LLC-Br cell line

**Fig. 1:**
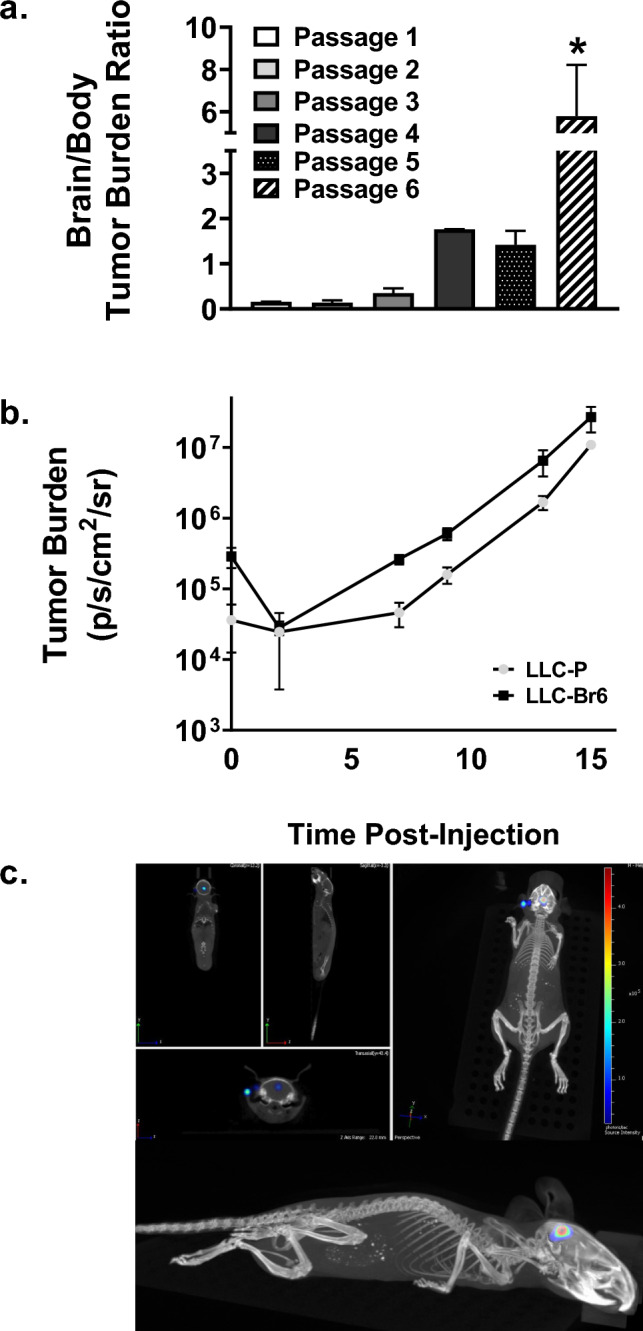
Tumor burden in the brain increases with passages of LLC brain explants **a** The ratio of BLI (radiance) in the brain vs body of mice increases over passages of the LLC-Br cell line. Passage 6 has a significantly higher brain vs body ratio at D14 in comparison with all previous passages. **b** Comparison of the brain tumor growth kinetics of the parental LLC (LLC-P) to the brain-tropic (LLC-Br) cell line in vivo. Brain tumor burden as measured by BLI (radiance) plotted over time (days post-injection of LLC cells). No significant differences in growth kinetics are present. **c** A representative image depicting localization of tumor cells in the brain with 3D bioluminescent CT imaging. *N* = 3–5

Wild-type C57Bl/6 mice were injected intracardially with LLC cells and allowed to develop brain tumors, which were then excised and cultured ex vivo. This process was repeated for six passages. The ratio of bioluminescent signal (radiance) in the brain versus body increased with each passage, shown in Fig. 1A. Passage six had a significantly higher brain-to-body tumor burden ratio than previous passages, with a ratio of 5.8 ± 2.4, indicating a sufficient brain metastasis model. Cells isolated from mouse brains following passage six were used in further experiments and hereafter referred to as LLC-Br. Brain tumor growth kinetics of LLC-Br and the parental cell line, LLC-P, were monitored with bioluminescent imaging. Despite mice injected with LLC-Br cells exhibiting greater brain-specific tumor burden, we did not observe any differences in the in vivo brain tumor growth rates between LLC-Br and LLC-P cell lines, as shown in Fig. 1B. Future BLI data with the LLC-Br model are normalized to day 3 because the growth is linear after this time point. The presence of LLC-Br brain lesions with little to no peripheral tumors was confirmed with 3D bioluminescent CT imaging. A representative image is shown in Fig. 1C.

### LLC-Br cell line is more invasive than parental cell line

**Fig. 2:**
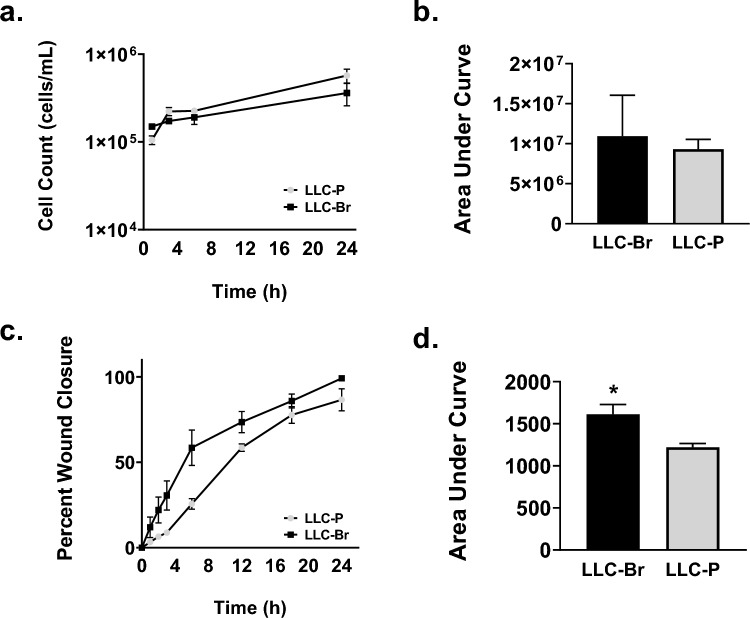
LLC-Br has a higher migration rate but similar growth rate compared to LLC-P **a** Comparison of the growth kinetics of LLC-Br and LLC-P over time as measured by counting in vitro cells at various time points. **b** No significant differences in cell growth rates as measured by area under the curve. **c** LLC-Br and LLC-P percent wound closure over time from in vitro scratch assay. **d** LLC-Br has significantly increased cell migration compared to the parental as measured by area under the curve. *N* = 6

Cells were counted at various time points over a period of 24 h to determine the growth rates of LLC-P and LLC-Br. Similar to in vivo findings above, LLC-P and LLC-Br growth rates did not differ significantly in vitro as measured by area under the curve (Fig. 2A–B). To evaluate the invasive capabilities of LLC-Br and LLC-P, a scratch assay was performed. We observed a significant increase in percent wound closure time in the LLC-Br cell line compared to LLC-P, as demonstrated by the area under the curve increasing from 1221 ± 44 to 1823 ± 115 (Fig. 2C–D).

### Similar tumor burden in wild-type and nude mice 22 days post-inoculation

**Fig. 3:**
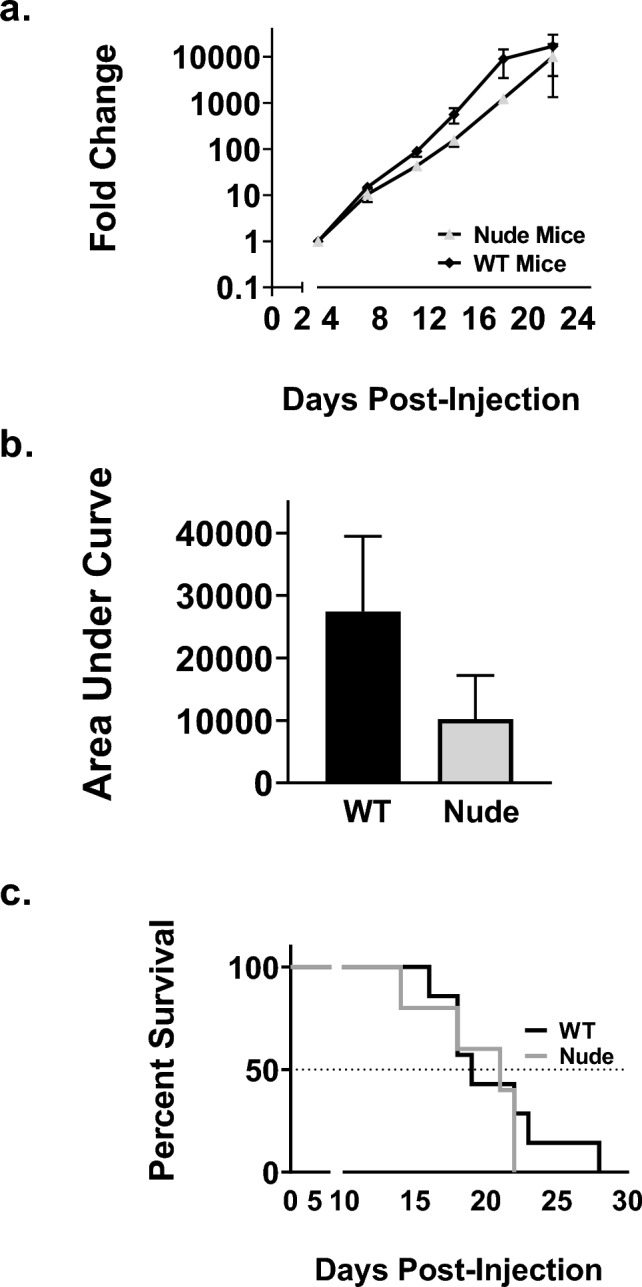
Similar tumor burden and survival in WT and nude mice **a** Tumor progression of LLC-Br in wild-type and athymic nude mice as measured by brain tumor growth via BLI over time normalized to day 3. **b** No significant differences in tumor burden between wild-type and nude mice as measured by area under the curve at day 22. **c** Kaplan–Meier plot of survival of WT and nude mice with LLC-Br tumors. No significant differences in median survival times. *N* = 7–9

The LLC-Br tumor progression with BLI in WT C57Bl/6 and nude mice was measured to confirm our model can be used in both mouse strains, as shown in Fig. 3. The fold change of LLC-Br brain tumor progression over time in each strain is shown in Fig. 3A. At day 22, we observed no differences in total brain tumor burden between nude mice (10,202 ± 6989) and WT mice (27,434 ± 12,040), shown in Fig. 3B. Additionally, we observed similar median survival between mouse strains, with nude mice having a median survival of 21 days compared to 19 days for WT mice (Fig. 3C).

### Temporal administration of immunotherapy affects brain tumor burden but not survival or weight loss

**Fig. 4:**
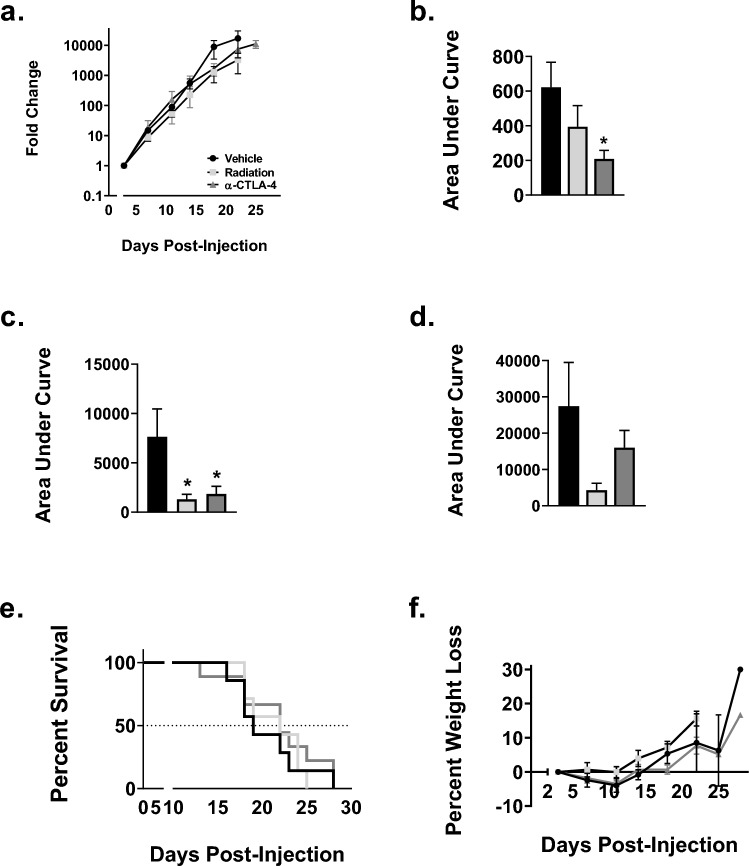
Delay in tumor progression until the conclusion of treatment in control groups. **a** Brain tumor progression of LLC-Br in wild-type mice treated with vehicle (solid black bar), radiation (solid light gray bar), or α-CTLA-4 (solid dark gray bar) as measured by bioluminescent imaging over time normalized to day 3. **b** Significant decrease in tumor burden of mice treated with α-CTLA-4 compared to vehicle at day 14. **c** Significant decrease in tumor burden of mice treated with radiation only or α-CTLA-4 only at day 18. **d** No significant differences in tumor burden between vehicle, radiation, and α-CTLA-4 groups as measured by area under the curve at day 22. **e** Kaplan–Meier plot of survival of WT mice with LLC-Br tumors treated with vehicle, radiation, or α-CTLA-4. **f** No significant differences in percent weight loss over time were observed. *N* = 7–9

**Fig. 5:**
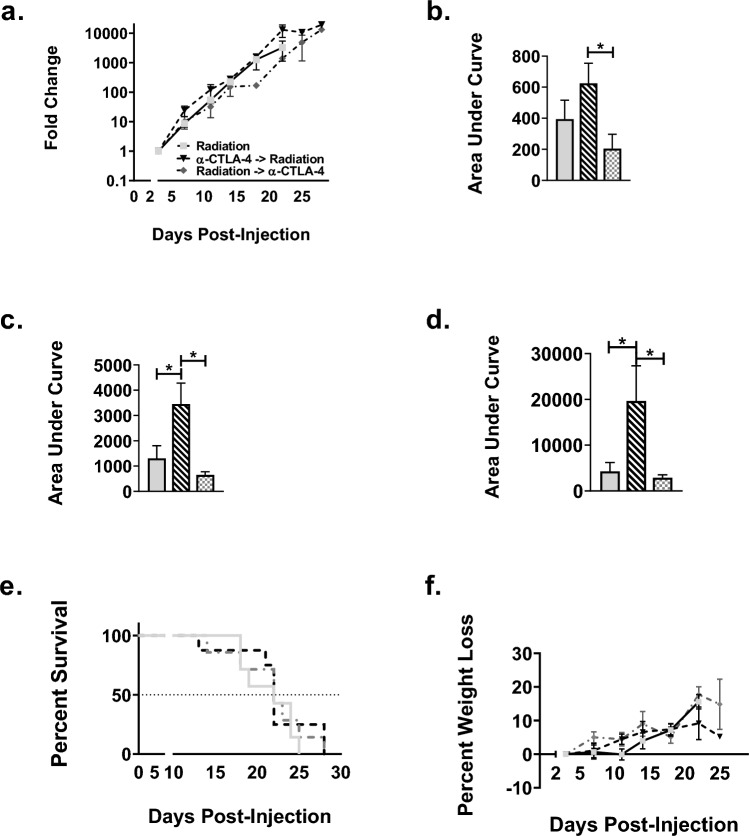
Potential tumor protective role of administering α-CTLA-4 24 h prior to whole-brain radiation therapy **a** Brain tumor progression of LLC-Br in wild-type mice treated with radiation (solid light gray bar), α-CTLA-4 24 h before radiation (black and white diagonal striped bar), or α-CTLA-4 12 h after radiation (gray and white checkered bar) as measured by bioluminescent imaging over time normalized to day 3. **b** Significant increase in tumor burden of mice treated with α-CTLA-4 24 h prior to radiation compared to 12 h after at day 14. **c** Significant increase in tumor burden of mice treated with α-CTLA-4 24 h prior to radiation at day 18. **d** Wild-type mice treated with α-CTLA-4 24 h before radiation had significantly higher tumor burden compared to radiation and α-CTLA-4 12 h after radiation groups as measured by area under the curve at day 22. **e** Kaplan–Meier plot of WT mice survival with LLC-Br tumors treated with radiation ± α-CTLA-4. **f** No significant differences in percent weight loss were observed in radiation groups. *N* = 7–9

**Fig. 6:**
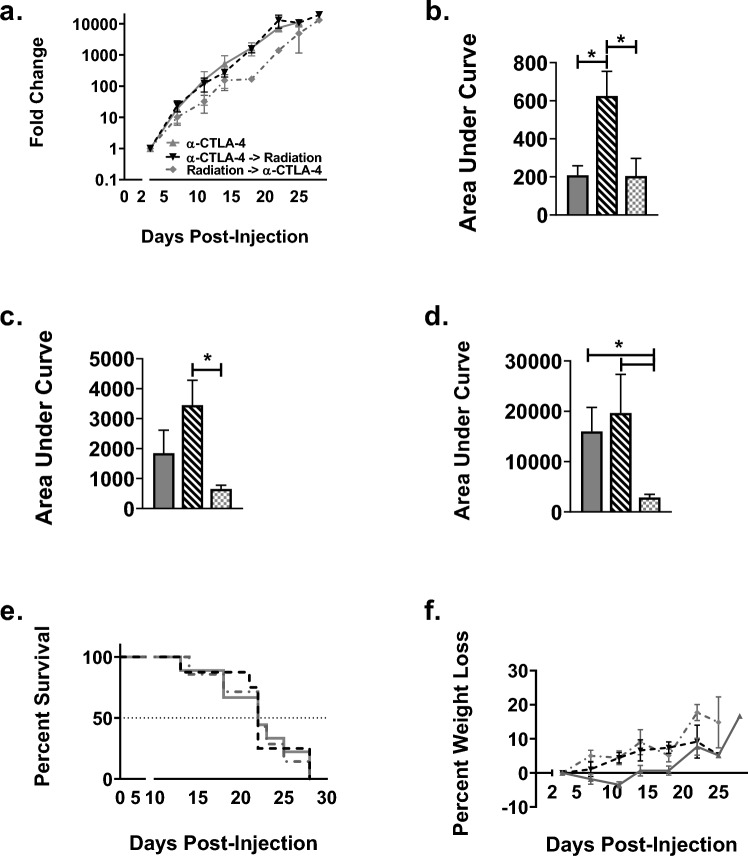
Administering α-CTLA-4 12 h post-radiation decreases tumor burden **a** Tumor progression of LLC-Br in wild-type mice treated with α-CTLA-4 only (solid dark gray bar), α-CTLA-4 24 h before radiation (black and white diagonal striped bar), or α-CTLA-4 12 h after radiation (gray and white checkered bar) as measured by bioluminescent imaging normalized to day 3. **b** Mice treated with α-CTLA-4 24 h prior to radiation had significantly higher brain tumor burden at day 14 compared to immunotherapy only or immunotherapy delivered 12 h post-WBRT. **c** Mice treated with α-CTLA-4 12-h post-WBRT had significantly lower brain tumor burden at day 18 than mice administered immunotherapy 24 h prior to radiation. **d** Wild-type mice treated with α-CTLA-4 12 h after radiation had significantly lower tumor burden compared to α-CTLA-4 only and α-CTLA-4 24 h before radiation groups as measured by area under the curve at day 22. **e** Kaplan–Meier plot of WT mice survival with LLC-Br tumors treated with α-CTLA-4 ± radiation. **f** No significant differences in percent weight loss were observed in the immunotherapy groups. *N* = 7–9

To examine the relationship between timing of immunotherapy administration and therapeutic response in combination with radiotherapy, we treated nude and WT mice bearing LLC-Br tumors with radiation therapy either 24 h after or 12 h before immunotherapy. After 14 days, tumor burden was significantly reduced in WT mice treated with either α-CTLA-4 alone (208 ± 50) or α-CTLA-4 administered 12-h post-WBRT (204 ± 93) groups compared to vehicle (622 ± 144) group (Figs. 4A–B, 5A–B, and 6A–B). At day 18, all treatment groups had significantly lower tumor burden compared to control (Figs. 4C, 5C, and 6C); however, by day 22, none of the treatment groups differed from vehicle mice (Figs. 4D, 5D, and 6D). Additionally, there were no significant differences observed in survival or weight loss (Figs. 4E–F, 5E–F, and 6E–F). All treatment groups had median survival times of 22 days compared to 19 days for the vehicle group.

A significant increase in tumor burden was observed in WT mice treated with α-CTLA-4 24 h prior to WBRT compared to mice treated with immunotherapy 12 h after radiation at days 14 (Fig. 5B), 18 (Fig. 5C), and 22 (Fig. 5D). Similarly, tumor burden was increased in mice treated with α-CTLA-4 prior to radiation compared to those treated with WBRT alone at days 18 (Fig. 5C) and 22 (Fig. 5D).

Temporal administration of immunotherapy with radiation altered treatment efficacy. The WT mice treated with α-CTLA-4 24 h prior to radiation had a higher tumor burden than mice treated with α-CTLA-4 only at day 14 (Fig. 6B). The group treated with α-CTLA-4 12 h after radiation had a significantly lower tumor burden at day 22 compared to the α-CTLA-4 only group (Fig. 6D).

As anticipated, the only treatment group with decreased tumor burden in nude mice was the radiation-only group at day 18 (Supp. Figure 1C). Surprisingly, this effect was absent at day 22 (Supp. Figure 1D). Similar to WT mice, no significant differences in survival or weight loss were observed between groups (Supp. Figures 1E–F, 2E–F, and 3E–F).

## Discussion

A major impediment to effective treatment of LCBM is the blood–tumor barrier (BTB), which restricts distribution of systemic therapeutic agents to lesions. The BTB tightly restricts access of conventional pharmacologic agents to CNS tumors, but immunotherapy presents a unique means to overcome this barrier to drug delivery by instead triggering activated immune cells to traverse the BTB and initiate destruction of intracranial tumor cells. For optimal immunotherapy efficacy, priming of the immune response and timing of administration are critical considerations. Without appropriate preclinical models of brain metastasis, the ramifications of sequencing immunotherapy with other local or systemic treatment modalities cannot be elucidated. Herein, we establish and characterize a novel syngeneic model of LCBM to evaluate the efficacy of immunotherapy and whole-brain radiation therapy.

We observed a significant increase in brain tumor burden compared to peripheral tumor burden upon the sixth passage of the LLC cell line, indicating a sufficient model to observe brain tumor-specific effects of treatment. Brain-specific tumor burden was confirmed with in vivo 3D bioluminescent imaging. As expected, the brain-tropic LLC-Br cell line exhibits increased motility compared to parental, LLC-P, cell line as measured by in vitro scratch assay. Additionally, we observed no differences in growth rates of LLC-P or LLC-Br cell lines in vitro or in vivo. Our model also has similar in vivo growth rates and survival times in WT and nude mice, suggesting that it is an appropriate model to compare the effects of the immune response on brain metastases and blood–tumor barrier.

Both radiation and α-CTLA-4 treatments resulted in significant decreases in tumor burden at days 14 and 18, but not at conclusion of the study (day 22). Unexpectedly, these treatments did not have an effect on survival or weight loss. We hypothesize that this could be occurring due to the rapid growth rate of LLC-Br cells. The treatments may delay tumor progression during and shortly following treatment, but lesions recur and mice succumb to the disease. This mimics clinical characteristics of LCBM, with 73–76% of patients treated with stereotactic radiosurgery (SRS) experiencing recurrence. When combined with WBRT, this decreases to 27–46% of patients [22]; however, toxicities associated with WBRT indicate an urgent need to identify systemic therapies for combination with SRS. Recurrence with immunotherapy is typically observed with α-PD-1; approximately 20% of patients with non-small cell lung cancer (NSCLC) initially respond to α-PD-1 treatment, but the majority develop resistance [23, 24].

We observed a significant increase in tumor burden in mice treated with α-CTLA-4 24 h before radiation, while there were no differences between mice treated with radiation alone and those treated with α-CTLA-4 12-h post-WBRT. A similar non-significant trend was observed in nude mice. Human-derived NSCLC cell lines express CTLA-4, and treatment with α-CTLA-4 antibody has been shown to induce PD-L1 expression. Additionally, binding of α-CTLA-4 promotes cell proliferation through activation of the EGFR pathway [25]. EGFR signaling is associated with diverse functions in lung cancer cells, including increased radioresistance, metastatic capabilities [26–28], DNA synthesis, proliferation, and cell cycle arrest [29]. It is well documented that phases of the cell cycle are a determinant of radiotherapy response, with cells in late S phase being most radioresistant and cells in M phase most radiosensitive [30, 31]. A study in 2020 demonstrated inhibition of EGFR/HER2 signaling in LLC cells results in decreased proliferation, reduced metastasis, and increased radiosensitivity [32]. We treated LLC-Br cells with α-CTLA-4 for 72 h and noted a significant increase in percent survival based on an MTT assay (Supp. Figure 4). Therefore, we hypothesize that the radioprotective effect we observed could be due to α-CTLA-4 stimulation of EGFR signaling, ultimately promoting proliferation, and potentially arresting cells in a more radioresistant phase of the cell cycle.

Between all immunotherapy groups, mice treated with α-CTLA-4 12 h post-radiation had the lowest tumor burden; conversely, the mice treated with α-CTLA-4 24 h prior to radiation had the highest tumor burden. These data suggest that timing of immunotherapy administration with radiotherapy does play a role in therapeutic efficacy and warrants further investigation. Treatment with α-CTLA-4 has varied effects depending on cell type and microenvironment [33], which could contribute to varied responses observed in immunotherapy studies.

Although we generated a syngeneic LCBM model and completed a study with immunotherapy and radiotherapy, our work has limitations. First, this study only evaluated a single low dose of α-CTLA-4 with radiotherapy. Future studies should evaluate if there is a dose effect of α-CTLA-4 when coordinated with WBRT. Additionally, we studied α-CTLA-4 as a single immunotherapy because we hypothesized that it would mobilize T-cells to brain lesions more effectively than α-PD-1 therapy. Studies to investigate coordination of α-CTLA-4 and α-PD-1 administration in combination with radiation should be performed. We hypothesize that combining α-PD-1 with α-CTLA-4 would enhance the synergistic effects we observed. Lastly, studies are needed to evaluate the mechanism of immunotherapy delaying progression with timed radiotherapy coordination.

## Conclusion

Patients with LCBM have poor prognosis and usually succumb to the disease within a year after diagnosis. Immunotherapy is a promising treatment modality for brain lesions, but preclinical models are limited. Our study demonstrates that we successfully created and characterized a syngeneic LCBM model to be used for immunotherapy studies. The model has a similar growth rate to its parental cell line, but higher motility. Additionally, we observed administration of α-CTLA-4 after radiation decreases brain tumor burden compared to α-CTLA-4 alone and administration of α-CTLA-4 prior to radiation. These data demonstrate the importance of optimizing sequence of treatment modalities and potentially increasing immunotherapy treatment throughout the course of disease to ensure positive outcomes. Further research is needed in the field of brain metastasis immunotherapy, and we aim to contribute with our novel LCBM model.

### Supplementary Information

Below is the link to the electronic supplementary material.

Supplementary file1 (EPS 63 KB)

Supplementary file2 (EPS 61 KB)

Supplementary file3 (EPS 60 KB)

Supplementary file4 (EPS 4 KB)

## Data Availability

The datasets used and/or analyzed for this study are available upon reasonable request from the corresponding author.
